# Effects of Repetitive Peripheral Magnetic Stimulation Versus Sham Stimulation on Upper Limb Spasticity After Stroke: A Double-Blind Randomized Controlled Trial

**DOI:** 10.3390/neurolint18090165

**Published:** 2026-08-27

**Authors:** Sasithorn Khawprapa, Nuttaset Manimmanakorn, Yohei Otaka, Jittima Saengsuwan

**Affiliations:** 1 Graduate School, Faculty of Medicine, Khon Kaen University, Khon Kaen 40002, Thailand; 2 Department of Rehabilitation Medicine, Graduate School of Medicine, Fujita Health University, Toyoake 470-1192, Aichi, Japan; 3 Department of Rehabilitation Medicine, Sakon Nakhon Hospital, Sakon Nakhon 47000, Thailand; 4 Department of Rehabilitation Medicine, Faculty of Medicine, Khon Kaen University, Khon Kaen 40002, Thailand; 5 Department of Rehabilitation Medicine, School of Medicine, Fujita Health University, Toyoake 470-1192, Aichi, Japan

**Keywords:** stroke, spasticity, peripheral magnetic stimulation, rehabilitation, shear wave elastography

## Abstract

**Background:** Upper limb spasticity after stroke occurs due to neural hyperexcitability and secondary alterations in muscle properties. Repetitive peripheral magnetic stimulation (rPMS) is a non-invasive technique used to reduce spasticity. This study aimed to compare the effects of rPMS versus sham stimulation on upper limb spasticity and muscle stiffness using shear wave elastography (SWE). **Methods:** This prospective, double-blind randomized controlled trial included 32 stroke patients with upper limb spasticity (Modified Ashworth Scale (MAS) ≥ 2 in the elbow flexors), who received rPMS or sham stimulation in addition to conventional rehabilitation (*n* = 16 per group). Spasticity was assessed using MAS, and muscle stiffness using SWE. **Results:** The rPMS group showed a transient short-term reduction in upper limb spasticity compared with the control group at the end of treatment (*p* = 0.045); however, this effect was not sustained at the 2-week follow-up. No significant between-group differences were observed in muscle stiffness, upper limb motor function, or activities of daily living. **Conclusions:** This study provides preliminary evidence that rPMS may produce a transient, short-term reduction in upper limb spasticity after stroke. However, this effect was not sustained at follow-up or accompanied by significant improvements in muscle stiffness or functional outcomes.

## 1. Introduction

Upper limb spasticity is common following a stroke, with the incidence varying according to time since onset [1,2,3,4]. As a component of upper motor neuron syndrome, its development involves not only reflex hyperexcitability but also maladaptive neural plasticity and secondary structural changes in muscles and soft tissues. Consequently, hypertonia comprises both reflex-mediated (spasticity) and non-reflex (intrinsic) components [5,6]. Clinically, spasticity most commonly affects upper limb flexor muscles, including the finger, wrist, and elbow flexors [6], and may lead to contractures, deformity, pain, and impaired movement, resulting in a reduced quality of life [7].

Various strategies are available for managing post-stroke upper limb spasticity, including physical therapy, orthotic devices, electrical stimulation, pharmacological treatments, and surgery. However, the responses to treatment vary between individuals [8,9,10,11,12,13,14,15]. Recently, repetitive peripheral magnetic stimulation (rPMS), a non-invasive technique that delivers magnetic pulses to peripheral nerves or muscles, has been increasingly recognized as a potential approach for reducing spasticity [16].

Previous studies have reported that rPMS may reduce post-stroke spasticity and improve upper limb motor function [17,18,19,20], possibly through modulation of cortical sensorimotor networks [21]. However, the findings have not been consistent across studies. Different stimulation approaches have been used, including combined transcranial and peripheral stimulation [17], peripheral intermittent theta-burst stimulation [18], and low-frequency rPMS targeting spastic muscles [20]. While these studies reported beneficial effects on spasticity, other studies using high-frequency rPMS with different stimulation targets did not demonstrate significant improvements in spasticity [22,23]. Variations in stimulation protocols may contribute to these conflicting findings. Thus, the clinical effectiveness and optimal application of rPMS for post-stroke spasticity remain unclear [24].

Shear wave elastography (SWE) is an ultrasound-based technique used to quantitatively assess muscle stiffness [25]; it measures tissue elasticity by analyzing shear wave propagation speed, with higher velocities indicating stiffer tissues. SWE has been used to evaluate muscle stiffness in patients with spasticity [26] and has shown good reproducibility [27]. Therefore, SWE may be useful for evaluating muscle stiffness in addition to clinical assessment in patients with post-stroke spasticity.

We hypothesized that rPMS combined with conventional rehabilitation would result in a greater reduction in upper limb spasticity than sham stimulation combined with conventional rehabilitation. Therefore, this study aimed to compare rPMS with sham stimulation in patients with stroke and to evaluate its effects on upper limb spasticity (primary outcome), muscle stiffness, and functional outcomes.

## 2. Materials and Methods

### 2.1. Study Design and Participants

This study was designed as a prospective, double-blind, randomized controlled trial with a 1:1 allocation ratio, conducted at the Department of Physical Medicine and Rehabilitation, Sakon Nakhon Hospital, Thailand. Participant recruitment and data collection were carried out between April 2025 and September 2025. The study protocol was approved by the Institutional Review Board of Sakon Nakhon Hospital (SKNHREC No. 058/2567) on 26 December 2024 and by the Center for Ethics in Human Research, Khon Kaen University (HE671390) on 17 September 2024. The trial was prospectively registered with the Thai Clinical Trials Registry on 2 March 2025 (registered number: TCTR20250302001; https://www.thaiclinicaltrials.org), where the trial protocol is publicly available. No important changes to the trial protocol were made after trial commencement. No patients or members of the public were involved in the design, conduct, reporting, or dissemination plans of this trial. The trial was completed as planned without early termination.

Patients were eligible for inclusion if they met all of the following criteria: (1) ischemic or hemorrhagic stroke confirmed via computed tomography or magnetic resonance imaging; (2) aged between 18 and 80 years; (3) upper limb spasticity, defined as a Modified Ashworth Scale (MAS) score ≥ 2 in the elbow flexors; (4) time since stroke between 2 weeks and 1 year; (5) no changes in antispastic medications within 1 month prior to recruitment. Patients were excluded if they met any of the following criteria: (1) presence of a cardiac pacemaker or a history of invasive cardiac procedures with unspecified details; (2) metallic implants in the affected upper limb; (3) pregnancy; (4) malignancy; (5) botulinum toxin injection to the affected upper limb within the previous 6 months; (6) inability to follow commands or the presence of cognitive impairment.

The sample size was calculated based on the primary outcome of upper limb spasticity measured using the MAS. Using a two-sided significance level of 0.05 and a statistical power of 80%, the calculation was based on a clinically meaningful difference of 0.4 and a standard deviation of 0.5, derived from a previous study [19]. The estimated sample size was 13 participants per group. To allow for potential participant dropout during follow-up, the target sample size was increased to 16 participants per group. No interim analyses or stopping guidelines were planned because of the short intervention period.

### 2.2. Randomization and Blinding

Eligible participants were enrolled by the principal investigator. The random allocation sequence was generated using a computer-based randomization program by an independent research assistant with no involvement in recruitment or assessment. Block randomization with a block size of 4 was used to ensure balanced allocation. Allocation was concealed using consecutively numbered sealed opaque envelopes, which were opened after baseline evaluation. Group allocation was implemented by an independent research assistant after baseline assessment, and participants were randomized to receive either rPMS or sham stimulation.

Participant blinding was maintained by using prerecorded rPMS sounds and minimal output (1%) to simulate active stimulation in the sham condition. Outcomes were assessed by a blinded physiatrist.

### 2.3. Intervention

#### 2.3.1. Intervention Group

Participants in the intervention group received rPMS using a MagRex electromagnetic stimulator (Medical Revolution Co., Ansan-si, Gyeonggi-do, Republic of Korea) with a 170 mm circular coil.

For each participant, the target muscles were selected according to the individual upper limb spasticity pattern. Stimulation was applied to the identified spastic muscles and their corresponding antagonists, defined as muscles producing the opposite movement at each joint. Depending on the individual spasticity pattern, the targeted muscle groups included the shoulder adductors and abductors, elbow flexors and extensors, and wrist flexors and extensors. The cumulative number of stimuli delivered per session was standardized across participants: spastic muscles received a total of 750 stimuli (5 Hz, 15 stimuli per train), whereas antagonist muscles received a total of 5100 stimuli (20 Hz, 30 stimuli per train). A 1 s inter-train interval was used, following a previously published protocol [19] in which low-frequency stimulation was applied to spastic muscles to reduce hyperexcitability, while higher-frequency stimulation was applied to antagonist muscles to facilitate motor activation and reciprocal inhibition [19,21]. The stimulation intensity was individually adjusted to a level just above the minimum motor threshold required to elicit a clearly visible contraction of the target muscle and remained above the motor threshold throughout the intervention sessions. Each session lasted approximately 30 min, and was followed by 20 min of conventional rehabilitation, consisting of individualized upper limb exercises, stretching, strengthening, and task-oriented functional training. Treatment sessions were conducted three times per week for two weeks. The interventions were delivered by trained physical therapists at Sakon Nakhon Hospital.

#### 2.3.2. Control Group

Participants in the control group received sham stimulation using the same device, coil placement, stimulation frequency, and number of stimuli as the intervention group, but with an output intensity of 1% of the maximum, producing an auditory cue without inducing muscle contraction. Each session lasted 30 min, followed by 20 min of conventional rehabilitation identical to that provided in the intervention group. Treatment sessions were conducted three times per week for two weeks. Adverse events related to rPMS or sham stimulation were monitored throughout the intervention period.

### 2.4. Measurements

#### 2.4.1. Primary Outcome Measure

Upper limb spasticity was assessed using the MAS, which ranges from 0 (no increase in muscle tone) to 4 (rigidity in flexion or extension), with higher scores indicating greater spasticity [28]. Spasticity was defined as an MAS score ≥ 2 [29]. MAS assessment for primary outcome was performed specifically for the elbow flexors of the affected upper limb; elbow flexor spasticity is a predominant manifestation after stroke and contributes to functional impairment of the upper limb [30,31].

#### 2.4.2. Secondary Outcomes Measures

Muscle stiffness was assessed using shear wave elastography (SWE), a quantitative ultrasound technique that estimates tissue stiffness based on the shear wave velocity, which is expressed as Young’s modulus in kilopascals (kPa); higher kPa values indicate increased muscle stiffness [32,33]. SWE measurements were performed on the biceps brachii muscle using a diagnostic ultrasound system (Aplio a550, Canon Medical Systems Corporation, Otawara, Japan), which has demonstrated good intra-rater reliability [34]. Participants were placed into a supine position, with the affected upper limb relaxed, the elbow maintained at a fully extended position, and the forearm supinated. Measurements were obtained after a 5 min rest period with the ultrasound probe aligned parallel to the muscle fibers over the distal third of the anterior arm [35].

Functional independence in activities of daily living was assessed using the Barthel Index, with total scores ranging from 0 to 100, with higher scores indicating more independence [36]. Upper limb motor function was assessed using the Fugl–Meyer Assessment—Upper Extremity (FMA-UE), which consists of 33 items with a maximum score of 66 points, with higher scores indicating better motor recovery [37,38].

Demographic data such as age, gender, and severity of stroke were recorded. All outcomes were measured before the rehabilitation program, at the end of the 2-week treatment period, and at the 2-week follow-up.

### 2.5. Statistical Analysis

The baseline characteristics were summarized using means and standard deviations for continuous variables and numbers with percentages for categorical variables. The primary outcome—elbow flexor spasticity assessed using the MAS—was analyzed according to the intention-to-treat principle using a proportional-odds mixed model to account for the ordinal nature of the MAS. Baseline MAS was included as a covariate to adjust for baseline differences between groups. Missing outcome data were handled using multiple imputation. A per-protocol analysis was additionally performed as a sensitivity analysis. Secondary outcomes, including muscle stiffness measured via SWE, upper limb motor function assessed via the FMA-UE, and functional independence assessed via the Barthel Index, were analyzed using linear mixed-effects models. An exploratory analysis examined the association between baseline SWE and time since stroke using Spearman’s rank correlation and multivariable linear regression adjusted for baseline MAS. All analyses were performed using Stata version 18 (StataCorp, College Station, TX, USA).

## 3. Results

### 3.1. Baseline Characteristics of Participants

**Figure 1:**
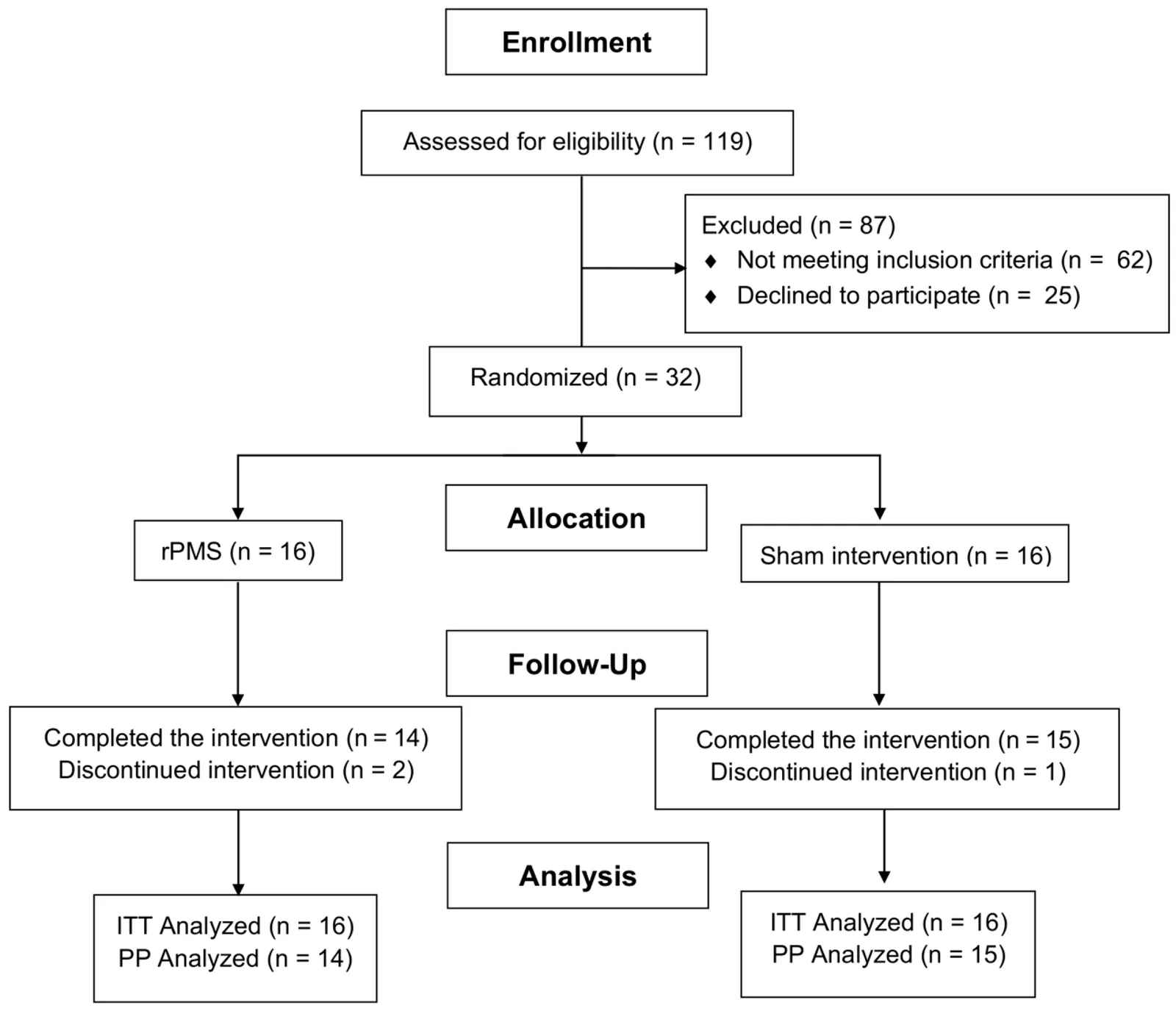
CONSORT flow diagram.

A total of 119 patients were screened for eligibility, of whom 32 were randomized to the rPMS group (*n* = 16) or the sham group (*n* = 16). Three participants discontinued the intervention due to transportation issues; however, all randomized participants were included in the ITT analysis (Figure 1). All participants in the rPMS group received low-frequency stimulation (5 Hz) to the elbow flexors and high-frequency stimulation (20 Hz) to the corresponding elbow extensors. No serious adverse events related to rPMS or sham stimulation were observed during the study period. The baseline demographic and clinical characteristics were comparable between groups. The mean age of the participants was 57.9 (SD = 9.5) years in the rPMS group and 59.25 (SD = 8.5) years in the control group. The mean time since stroke was 24.9 weeks (SD = 13.5; range, 8–48 weeks) in the rPMS group and 23 weeks (SD = 11.8; range, 8–44 weeks) in the control group. Other baseline characteristics are presented in Table 1, with no significant differences observed between groups at study entry.

### 3.2. Primary Outcome: Elbow Flexor Spasticity

At the end of the 2-week treatment period, the rPMS group showed a significantly greater reduction in spasticity compared with the control group (*p* = 0.045). A decrease of at least one grade on the MAS from baseline was observed in 10 of 14 participants (71.4%) in the rPMS group and 3 of 15 participants (20.0%) in the control group; this between-group difference was not statistically significant at the 2-week follow-up (*p* = 0.363). Within-group analysis also demonstrated significant improvements in MAS scores in the rPMS group from baseline to the end of treatment (*p* < 0.05) (Table 2).

A PP analysis was conducted as a sensitivity analysis, including participants who completed the intervention and had complete post-intervention and follow-up data. The PP analysis also demonstrated a significant difference between the two groups at post-intervention (*p* = 0.036), consistent with the ITT findings, while no significant between-group difference was observed at the follow-up (*p* = 0.415). Overall, the direction of the treatment effects was consistent between the ITT and PP analyses (Table 3). Model fit was assessed using the Akaike Information Criterion (AIC) and Bayesian Information Criterion (BIC). The proportional-odds mixed-effects model yielded an AIC of 75.50 and a BIC of 89.93.

### 3.3. Secondary Outcomes

No statistically significant adjusted mean differences were observed in biceps muscle stiffness, measured using SWE, at the end of the treatment period (*p* = 0.851) or at the follow-up (*p* = 0.827) (Table 4). The within-group analysis also showed no significant changes in the SWE (all *p* > 0.05). Additionally, no significant between-group differences were found in the upper limb motor function assessed using the FMA-UE and the activities of daily living assessed using the Barthel Index at any time point (all *p* > 0.05). However, the within-group analysis showed a significant improvement in the Barthel Index scores at follow-up compared with the baseline in both the rPMS (*p* = 0.004) and control (*p* = 0.006) groups. In an exploratory analysis, Spearman’s rank correlation showed no significant association between time since stroke and baseline SWE (ρ = 0.252, *p* = 0.165). However, in the multivariable linear regression model adjusted for baseline MAS, time since stroke was significantly associated with baseline SWE (β = 0.343, 95% CI 0.075–0.611, *p* = 0.014) (Table 5).

## 4. Discussion

Our findings suggest that rPMS may produce a transient short-term reduction in upper limb spasticity, which is consistent with previous randomized controlled trials reporting its efficacy across a range of stimulation protocols and parameters [18,19,20]. Similar findings were observed in both the intention-to-treat and per-protocol analyses, supporting the consistency of the observed treatment effect. However, there were no significant differences in SWE and functional outcomes between the two groups.

Previous studies have shown significant reductions in spasticity using different stimulation approaches. Werner et al. (2016) observed immediate reductions in muscle tone following a single 5 Hz stimulation session [20]. Chen et al. (2020) reported significant reductions in MAS scores following rPMS using a 5 Hz protocol combined with 20 Hz stimulation of antagonist muscles [19]. Similarly, Nahas et al. (2022) found that theta-burst stimulation, consisting of bursts delivered at 50 Hz and repeated at a theta frequency of 5 Hz, resulted in a significant reduction in spasticity compared with sham stimulation [18]. Our findings are consistent with these studies, demonstrating that 5 Hz stimulation combined with higher-frequency stimulation (20 Hz) of antagonist muscles can effectively reduce short-term spasticity.

Physiological evidence has shown that low-frequency rPMS (5 Hz) can reduce tendon reflex activity, reflecting decreased spinal reflex excitability and reduced muscle tone [39]. In addition, neurophysiological studies have shown that a single session of rPMS is associated with changes in cortical activity on electroencephalography (EEG), suggesting that rPMS may induce cortical neuromodulation [19]. Together, these previous findings suggest possible spinal and cortical mechanisms.

The antispastic effects of rPMS were not sustained at the 2-week follow-up in this study; these findings are consistent with previous studies. Krewer et al. [40] demonstrated short-term reductions in wrist flexor spasticity, whereas longer-term effects were observed only in specific muscle groups, such as elbow extensors. Notably, the long-term differences in elbow extensors may be due to an increase in spasticity in the control group, rather than a therapeutic effect in the rPMS group. However, the relatively short follow-up period remains a limitation of the present study, and longer follow-up would provide a more complete evaluation of the long-term effects of rPMS. Whether different stimulation frequencies and treatment doses could prolong these effects also requires further investigation.

Our study found no significant between-group differences in functional outcomes, including the FMA-UE and the Barthel Index. In contrast, Fawaz et al. (2023) demonstrated significant improvements in both motor function and spasticity following rPMS compared with the control group after 3 weeks of treatment [41]. These inconsistencies may be attributed to differences in stimulation protocols, including the use of higher-frequency stimulation (30 Hz) targeting antagonist muscles opposing the flexor synergy pattern, longer train durations, and a more intensive treatment schedule (five sessions per week for three weeks), resulting in a higher cumulative stimulation dose than in the present study. In this study, both groups showed significant improvement in the Barthel Index at the 2-week follow-up compared with baseline, likely reflecting the effect of the conventional rehabilitation program provided to both groups.

Muscle stiffness, as assessed via SWE, showed no significant between-group differences at either post-treatment or the 2-week follow-up. The reduction in MAS scores without a corresponding change in SWE may reflect an effect on neural mechanisms rather than changes in the mechanical properties of the muscle [19,21]. However, neural mechanisms were not directly assessed in this study. The relatively short 2-week intervention period may also have been insufficient to induce measurable changes in muscle stiffness. Furthermore, because SWE was evaluated under static conditions in our study, dynamic SWE during passive movement at different velocities may provide additional information on velocity-dependent changes in muscle stiffness associated with spasticity [42,43].

To investigate factors associated with baseline muscle stiffness, we performed additional analyses. Although Spearman’s rank correlation was not statistically significant, multivariable linear regression demonstrated a significant association between time since stroke and baseline SWE after adjustment for baseline MAS. However, the underlying mechanism remains unclear, and these findings should be interpreted with caution because the analysis was not prespecified and the study was not designed or powered to evaluate factors associated with baseline SWE.

The present study extended a previously reported single-session rPMS protocol [19] to a 2-week repeated intervention. While the previous study demonstrated an immediate reduction in spasticity after a single session, we evaluated the effects of repeated rPMS both post-intervention and at a 2-week follow-up. SWE was also included to examine the relationship between clinical spasticity and muscle stiffness.

From a clinical perspective, reducing spasticity may reduce resistance to movement and facilitate stretching and functional training [44,45]. However, the transient reduction in MAS observed in the present study was not accompanied by significant improvements in functional outcomes, suggesting that spasticity reduction alone may not be sufficient to improve function [9,46]. Further studies are needed to determine whether combining rPMS with task-specific training and strengthening exercises can improve functional recovery [47,48].

## 5. Study Limitations

The sample size was calculated based on the primary outcome of spasticity severity and may have been insufficient to detect differences in secondary outcomes, including muscle stiffness assessed by SWE and functional outcomes. The evaluation of treatment effects was also limited by assessing spasticity only at the elbow flexors, despite applying rPMS to multiple upper limb muscle groups. In addition, lesion location and sensory impairments were not systematically collected, although these factors may influence upper limb motor recovery and responsiveness to rPMS. Objective neurophysiological assessments were not performed, limiting interpretation of the proposed neural mechanisms. Because active rPMS produced visible muscle contractions, some participants may have inferred their group allocation, potentially affecting participant blinding.

## 6. Conclusions

This study provides preliminary evidence that rPMS may produce a transient, short-term reduction in upper limb spasticity after stroke. However, this effect was not sustained at the 2-week follow-up and was not accompanied by significant improvements in muscle stiffness or functional outcomes. Larger, adequately powered randomized controlled trials are needed to confirm these findings.

## Figures and Tables

**Table 1:** Baseline characteristics.

Characteristic	rPMS (*n* = 16)	Control (*n* = 16)	*p*-Value
Sex, *n* (%)			0.264
Male	12 (75.0)	9 (56.3)	
Female	4 (25.0)	7 (43.7)	
Age(year), mean (SD)	57.9 (9.5)	59.25 (8.5)	0.683
Stroke type, *n* (%)			0.063
Ischemic stroke	8 (50.0)	13 (81.2)	
Hemorrhagic stroke	8 (50.0)	3 (18.7)	
Time since stroke in weeks, mean (SD)	24.9 (13.5)	23 (11.8)	0.669
Hemisphere of lesion, *n* (%)			0.719
Left	7 (43.8)	6 (37.5)	
Right	9 (56.2)	10 (62.5)	
Antispastic drug use, *n* (%)	8 (50.0)	7 (43.7)	0.723
Hand splint use, *n* (%)	1 (6.2)	3 (18.7)	0.285

**Abbreviations:** rPMS, repetitive peripheral magnetic stimulation.

**Table 2:** Comparison of Modified Ashworth Scale (MAS) at the elbow flexors between groups (intention-to-treat analysis).

Outcome	Time Point	rPMS (*n* = 16) Median (IQR)	Control (*n* = 16) Median (IQR)	OR (95% CI)	*p*-Value
MAS (Elbow flexors)	Baseline	3 (2–3)	2 (2–3)	-	0.724
	Week 2 (end of treatment)	2 (1.5–2) *	2 (2–3)	0.042 (0.002–0.930)	0.045 **^†^**
	Week 4 (2-week follow-up)	3 (2–3)	2 (2–3)	0.318 (0.027–3.756)	0.363
Overall group × time interaction					0.135

**Abbreviations:** IQR, interquartile range; rPMS, repetitive peripheral magnetic stimulation. **Note:** * *p* < 0.05 (within-group). ^†^ Between-group comparisons were performed with a proportional-odds mixed model.

**Table 3:** Sensitivity analysis of between-group comparisons for Modified Ashworth Scale (MAS) at the elbow flexors using intention-to-treat (ITT) and per-protocol (PP) analyses.

Outcome	Time Point	ITT (*n* = 32) OR (95% CI)	ITT (*n* = 32) *p*-Value	PP (*n* = 29) OR (95% CI)	PP (*n* = 29) *p*-Value
MAS (Elbow flexors)	Baseline	-	0.724	-	0.582
	Week 2 (end of treatment)	0.042 (0.002–0.930)	0.045	0.039 (0.002–0.804)	0.036
	Week 4 (2-week follow-up)	0.318 (0.027–3.756)	0.363	0.368 (0.033–4.076)	0.415
Overall group × time interaction		-	0.135	-	0.182

**Note:** *p*-values in both columns were derived from a proportional-odds mixed model.

**Table 4:** Secondary outcomes over time in the repetitive peripheral magnetic stimulation (rPMS) and control groups.

Outcome	Time Point	rPMS (*n* = 16) Estimated Mean (SE)	Control (*n* = 16) Estimated Mean (SE)	Adjusted Mean Difference (95% CI)	*p*-Value ^†^
SWE in the biceps brachii, kPa	Baseline	69.73 (4.14)	66.79 (4.14)	2.94 (−8.91–14.78)	0.627
	Week 2 (end of treatment)	67.38 (4.17)	64.85 (4.15)	−0.41 (−4.67–3.85)	0.851
	Week 4 (2-week follow-up)	68.27 (4.17)	65.81 (4.15)	−0.47 (−4.73–3.79)	0.827
FMA-UE	Baseline	15.94 (2.58)	15.06 (2.58)	0.87 (−6.27–8.02)	0.810
	Week 2 (end of treatment)	16.08 (2.58)	15.13 (2.58)	0.95 (−6.19–8.10)	0.794
	Week 4 (2-week follow-up)	16.16 (2.58)	15.33 (2.58)	0.83 (−6.32–7.98)	0.821
Barthel Index	Baseline	58.12 (7.39)	49.38 (7.39)	8.75 (−11.76–29.26)	0.403
	Week 2 (end of treatment)	58.50 (7.40)	49.70 (7.40)	8.79 (−11.72–29.31)	0.401
	Week 4 (2-week follow-up)	60.28 (7.40) *	51.37 (7.40) *	8.91 (−11.60–29.43)	0.395

**Abbreviations:** SWE, shear wave elastography; FMA-UE, Fugl–Meyer Assessment for Upper Extremity. **Notes:** * Within-group change from baseline (*p* < 0.05). ^†^ Between-group comparisons were performed with linear mixed-effects models.

**Table 5:** Exploratory analyses of the association between time since stroke and baseline muscle stiffness assessed via shear wave elastography.

Analysis/Variable	Estimate	95% CI	*p*-Value
Baseline SWE vs. time since stroke			
Unadjusted model	0.252	-	0.165
Adjusted model ^a^	0.343	0.075–0.611	0.014

**Abbreviations:** SWE, shear wave elastography. **Note:** ᵃ Adjusted for baseline Modified Ashworth using a multivariable linear regression model. The regression coefficient represents the estimated change in baseline SWE (kPa) for each one-week increase in time since stroke.

## Data Availability

The data underlying this study can be obtained from the corresponding author upon request.

## Abbreviations

rPMS	repetitive peripheral magnetic stimulation
SWE	shear wave elastography
MAS	Modified Ashworth Scale
FMA-UE	Fugl–Meyer Assessment Upper Extremity
ITT	intention-to-treat
PP	per-protocol
EEG	electroencephalography
SD	standard deviation
IQR	interquartile range
CI	confidence interval
kPa	kilopascal
